# Bone transport combined with bone graft and internal fixation versus simple bone transport in the treatment of large bone defects of lower limbs after trauma

**DOI:** 10.1186/s12891-022-05115-0

**Published:** 2022-02-17

**Authors:** Qiang Huang, Yi Bo Xu, Cheng Ren, Ming Li, Cheng Cheng Zhang, Lu Liu, Qian Wang, Yao Lu, Hua Lin, Zhong Li, Han Zhong Xue, Kun Zhang, Teng Ma

**Affiliations:** Department of Orthopaedic Surgery, Hong Hui hospital, Xi’an Jiaotong University College of Medicine, Xi’an, 710054 Shaanxi China

**Keywords:** Bone transport, Bone graft, Internal fixation, Bone defect

## Abstract

**Background:**

Bone transport has been successfully applied for the management of large segmental bone defects. However, its main shortcoming is the long-lasting consolidation period, which may cause lots of related complications. To overcome this shortcoming, we developed bone transport combined with bone graft and internal fixation technique. The purpose of this study was to compare the clinical effects of this modified technique with simple bone transport in the treatment of large segmental bone defects of lower limbs after trauma.

**Methods:**

Eighty-four patients with large segmental bone defects treated in our institution from January 2014 to January 2017 were selected for retrospective study. A total of 77 cases were completely followed. Among them, 35 patients were treated by bone transport combined with bone graft and internal fixation technique (Group A), and 42 by simple bone transport technique (Group B). Patients with open injuries were classified according to Gustilo-Anderson (GA) classification. The general data of Group A and B were compared. The time in external fixator, total cure time and operation times of two groups were recorded. Ennecking score was used to evaluate the recovery of limb functions while self-rating anxiety scale (SAS) for the postoperative anxiety evaluation. In addition, the total complication incidence was compared between Group A and B.

**Results:**

There was no significant difference in demographic data between Group A and B (*p* > 0.05). The time in external fixator of Group A and B was (4.8 ± 1.6) and (18.2 ± 3.9) months, respectively (*p* < 0.05). The total cure time was (17.6 ± 2.2) and (20.4 ± 2.8) months in Group A and B (*p* < 0.05). The number of operations in Group A and B was (4.9 ± 1.2) and (4.8 ± 1.0) (*p* > 0.05). Ennecking score of Group A and B was 84.7 and 75.7% (*p* < 0.05). SAS score and total complication incidence in Group A were significantly lower than those in Group B (*p* < 0.05).

**Conclusions:**

The clinical effects of bone transport combined with bone graft and internal fixation technique were better than that of simple bone transport technique, including shorter time in external fixator, shorter total cure time, lower anxiety score and better limb functions.

## Background

The post-traumatic bone defects are mostly caused by high-energy injuries, such as traffic accidents, falling height, heavy pound injury, etc. Infection after internal fixation can also lead to the formation of large segmental sequestrum, and obvious bone defects will form after debridement. Due to long cycle and lots of complications, it’s difficult to treat such patients. As a result, it brings great economic, psychological and social pressure to patients, and seriously affects their life quality. Surgical intervention is the main measure to repair large segmental bone defects. At present, surgical methods for the treatment of long bone defects exceeding the critical-sized bone defects (5 cm) mainly include vascularized bone transplantation, Masquelet technique, bone transport and so on [1–4].

Bone transport technique is also called distraction osteogenesis technique. The wide application of this technique has saved many limbs on the verge of amputation. According to the tension-stress principle, it stimulates the regenerative potential of bone tissues through slow and continuous tensile stress, and the gap generated by traction will be repaired by new callus. Meanwhile, the surrounding vessels, nerves, fascia and muscles will grow synchronously [5]. The bone transport frame can be fixed away from injured region or infection area. In this way, the interference to soft tissues is small. It can not only repair large segmental bone defects, but also correct limb shortening and deformity [6]. Although bone transport is effective in the treatment of large segmental bone defects, it also has some obvious shortcomings. First of all, patients have to tolerate a long-lasting consolidation period [7]. Using bone transport technique, it takes more than 30 days to repair one-centimeter bone defect on average. During this process, the psychological burden of these patients increases while the life quality decreases. Moreover, the long-lasting consolidation period leads to many related complications, such as pin-tract infection, axial deviation, docking site nonunion, joint stiffness, horseshoe varus, new callus deformation and re-fracture at the regenerate site, etc. [3, 4, 8].

Autologous bone graft technique is also a common method for the treatment of large segmental bone defects. It repairs bone defects through bone conduction, induction and osteogenesis. It is the golden standard for bone graft because of good histocompatibility and no immune rejection after transplantation [9]. The most common autologous bones used by trauma surgeons are iliac bones. Although autologous bone graft is effective, the size and quantity are limited, and the amount varies due to the individual differences of every patient [10, 11]. For large segmental bone defects that are greater than the critical-sized bone defects (5 cm), there will be uncontrollable bone necrosis and resorption. Therefor, the curative effects of non vascularized autologous bone graft are not accurate [12–15].

**Fig. 1:**
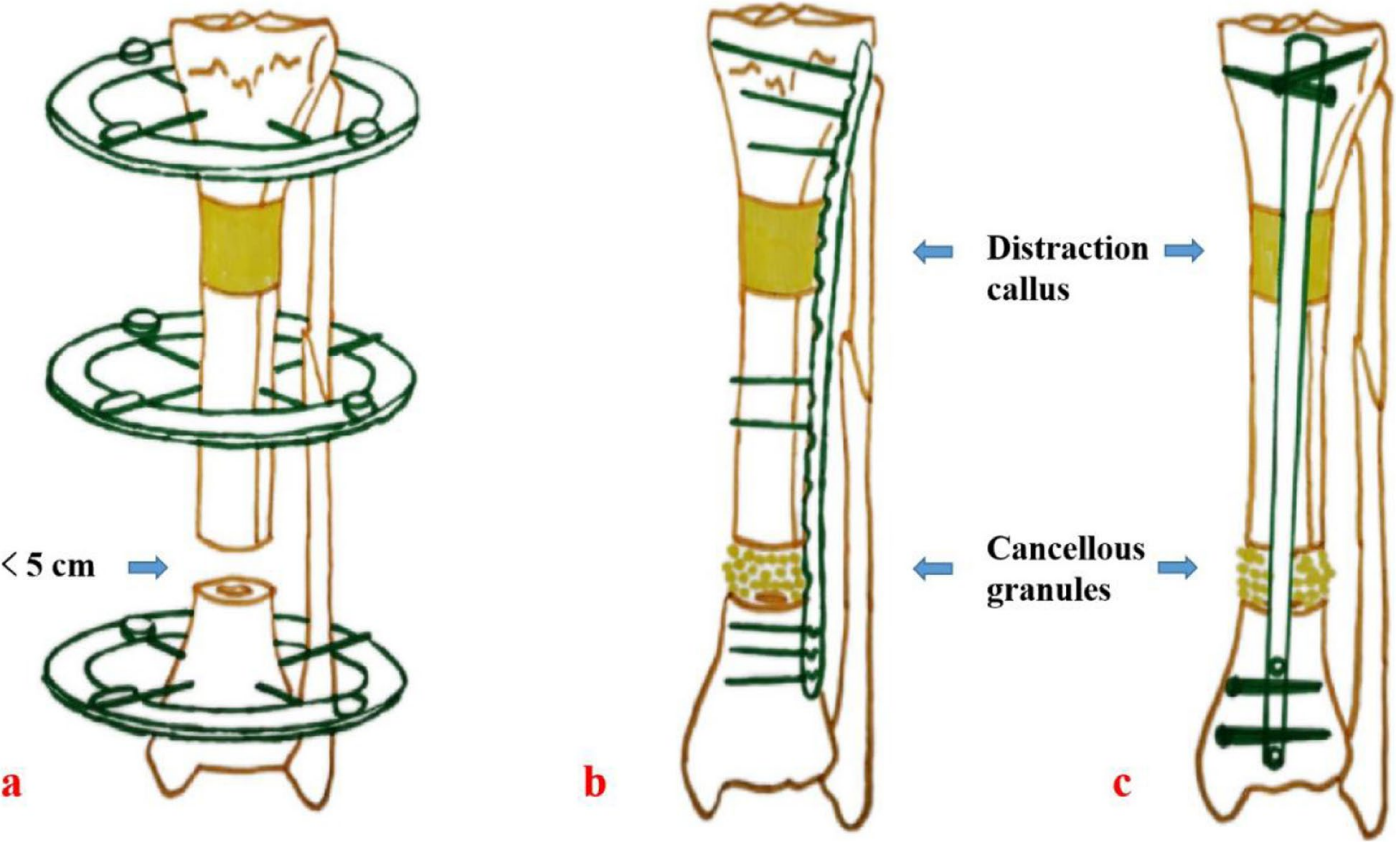
Schematic diagram of bone transport combined with bone graft and internal fixation technique. a: At the initial stage, the bone defects were shortened to less than 5 cm by bone transport; b: After removing the transport frame, we replaced it with a long plate, and performed cancellous bone graft; c: After removing the transport frame, surgeons can also replace it with an intramedullary nail, and then perform cancellous bone graft

We combined bone transport with autologous bone graft and internal fixation to treat patients with large segmental bone defects (as shown in Fig. 1). That is, the bone defect length was first shortened to within the critical-sized bone defects by bone transport, usually 3–4 cm. Then, the transport frame was removed and converted to non vascularized autologous bone graft and internal fixation until the end. This study compared the clinical effects of our modified technique with simple bone transport technique in the treatment of large segmental bone defects. The details are as follows.

## Patients and methods

### Inclusion and exclusion criteria

Inclusion criteria: (1) Patients older than 18 years; (2) Patients with large segmental bone defects after trauma; (3) Patients with complete medical records; (4) Patients with bone defects longer than 8 cm; (5) Patients were treated by bone transport technique at the initial stage.

Exclusion criteria: (1) Patients younger than 18 years; (2) Patients with bone defects less than 8 cm; (3) Patients with non traumatic bone defects; (4) Patients with severe medical diseases unable to tolerate a surgery or anesthesia; (5) Passive removal of the transport frame due to poor osteogenesis or intolerance; (6) Patients with incomplete medical records.

### General data

Eighty-four patients with large segmental bone defects after trauma treated in our institution from January 2014 to January 2017 were selected, and a total of 77 cases were completely followed. There were 54 males and 23 females, aged 19–68 years. Thirty-five patients were treated by bone transport combined with bone graft and internal fixation technique (Group A), and 42 by simple bone transport technique (Group B). This study was approved by the ethics committee of Xi’an Hong Hui hospital (IRB approval number: 202104018). All patients or their families have signed the informed consent before operation.

Preoperative treatment: Patients needed a general examination after admission. The affected limb was routinely examined by X-ray and CT, and the infection-related indexes such as erythrocyte sedimentation rate (ESR) and C-reactive protein (CRP) were detected by blood sampling. For bone defects caused by an open fracture, damage control surgery should be performed first, including debridement and temporary fixation. For patients with chronic osteomyelitis, thorough debridement should also be performed, and sequestrum should be removed wholely. After infection was controlled and soft tissues healed, bone defects were repaired in time.

### Surgical procedures

The annular external fixator (Naton, China) or single-arm transport frame (Naton, China) was placed. The limb length was maintained through traction. Meanwhile, the alignment and rotation deformity were corrected. Parallel to the articular surface, the distal and proximal ends of the long bone were fixed on the frame, respectively. The transport pins were inserted at the appropriate position to fix the transport segment. According to the surgical design, osteotomy was performed at the distal or proximal part of the long bone. The limb length and alignment were confirmed by an image intensifier. Then, the wounds were rinsed and sutured. The bone transport process began 1 week after operation.

**Fig. 2:**
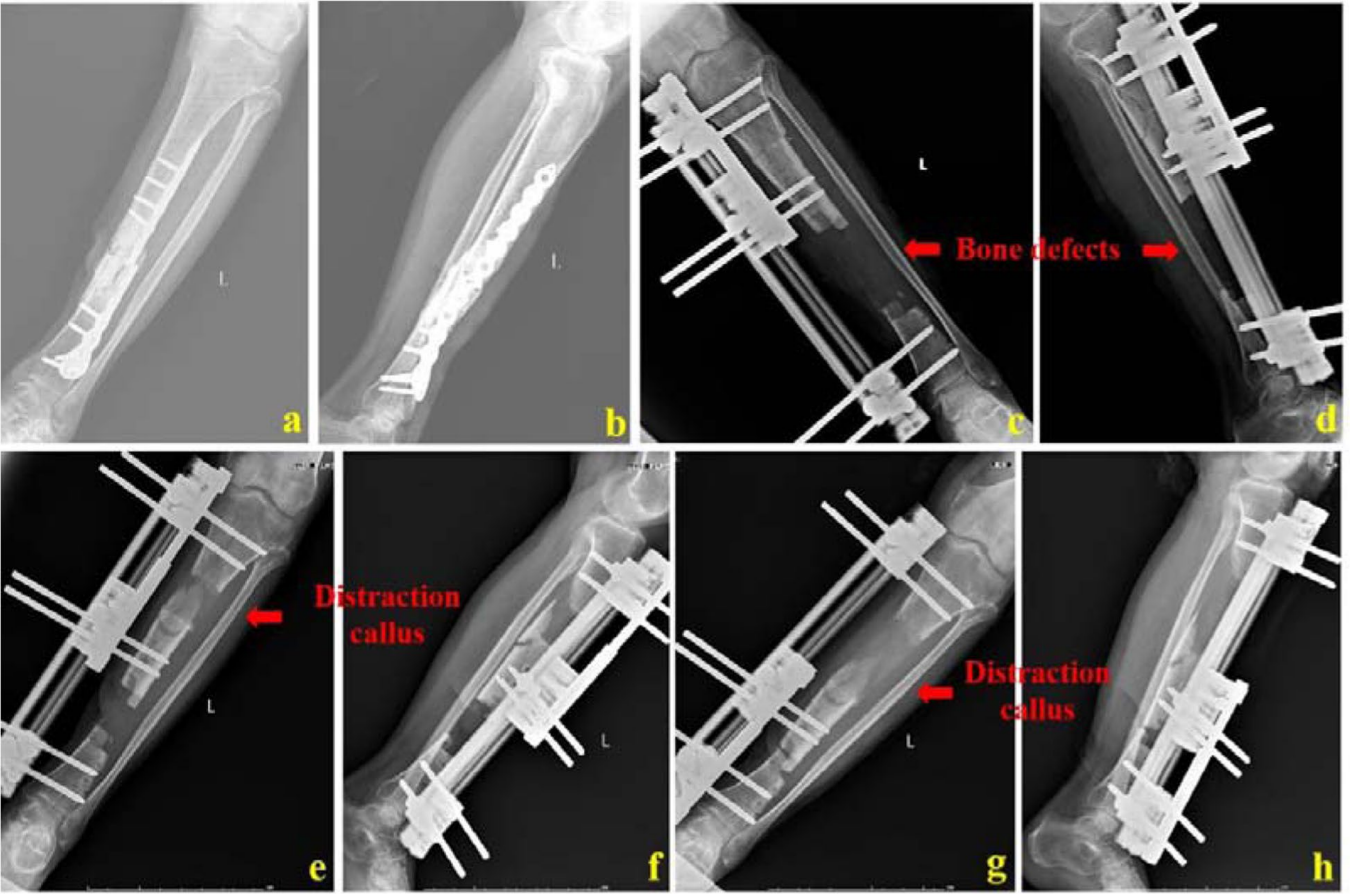
A 55-year-old male was successfully treated by bone transport combined with bone graft and internal fixation technique. a and b: The patient had infectious nonunion of tibia and the plate was broken; c and d: After the whole segment of sequestrum was cut, the bone defect length was 12 cm. At the initial stage, surgeons used a single-arm transport frame for bone transport; e and f: X-ray images when bone transport was performed for one and a half months; g and h: X-ray images when bone transport was performed for 3 months

**Fig. 3:**
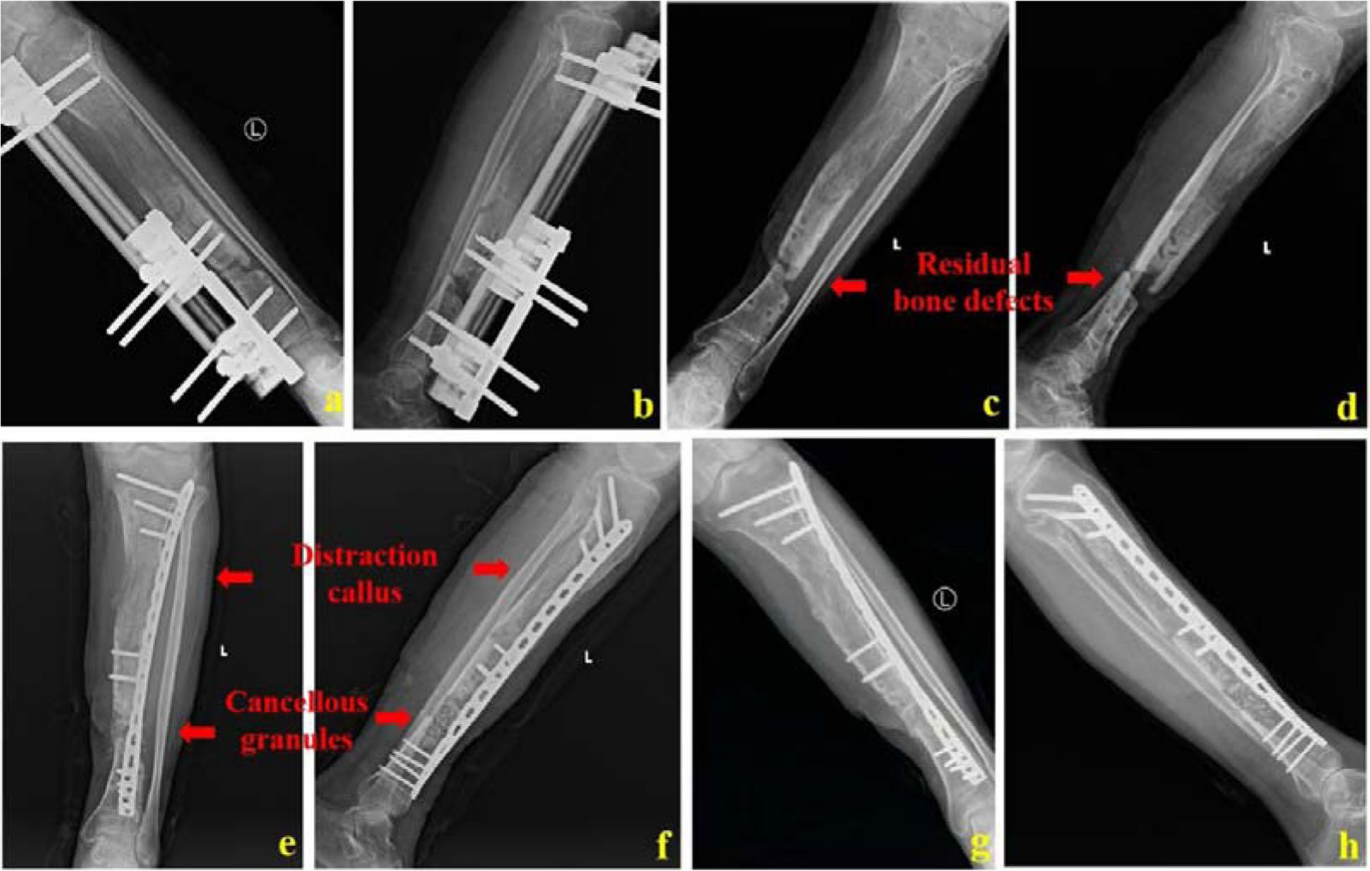
The 55-year-old male was treated by bone transport combined with bone graft and internal fixation technique. a and b: The bone defect length was shortened to 3.5 cm when the single-arm external frame was ready to be removed; c and d: X-ray images after removing the transport frame; e and f: X-ray images after cancellous bone graft and plate fixation; g and h: One year after bone graft, the bone defect healed well

For patients of Group A, when the bone defects were shortened to less than 5 cm, bone transport stopped. The transport frame was removed and the affected limb was fixed with a plaster or brace for 1 week. This would promote healing of the pin-tracts. After the pin-tracts healed, sequential bone graft and internal fixation started. With the residual bone defects as the center, a longitudinal incision was taken. The incision length was about 8–10 cm. It was cut layer by layer. The residual bone defects were exposed. Both ends of the bone defects were trimmed until there was good blood seepage on the bone surface. The scar tissues were removed, too. After thoroughly washing the wounds, a plate or intramedullary nail (Naton, China) shall be selected according to the preoperative design. The whole segment could be bridged by minimally invasive percutaneous placement of a plate. An intramedullary nail could also be placed. The distal and proximal ends of the intramedullary nail were locked, respectively. The residual bone defects were measured, the amount of autologous bones needed was roughly estimated, and the autologous iliac bones were taken and implanted into the residual bone defect site. Finally, a drainage tube was inserted and the incisions were closed. It was confirmed by an image intensifier that the transplanted bones were sufficient and the internal fixation position was good. A typical case was shown in Figs. 2 and 3.

**Fig. 4:**
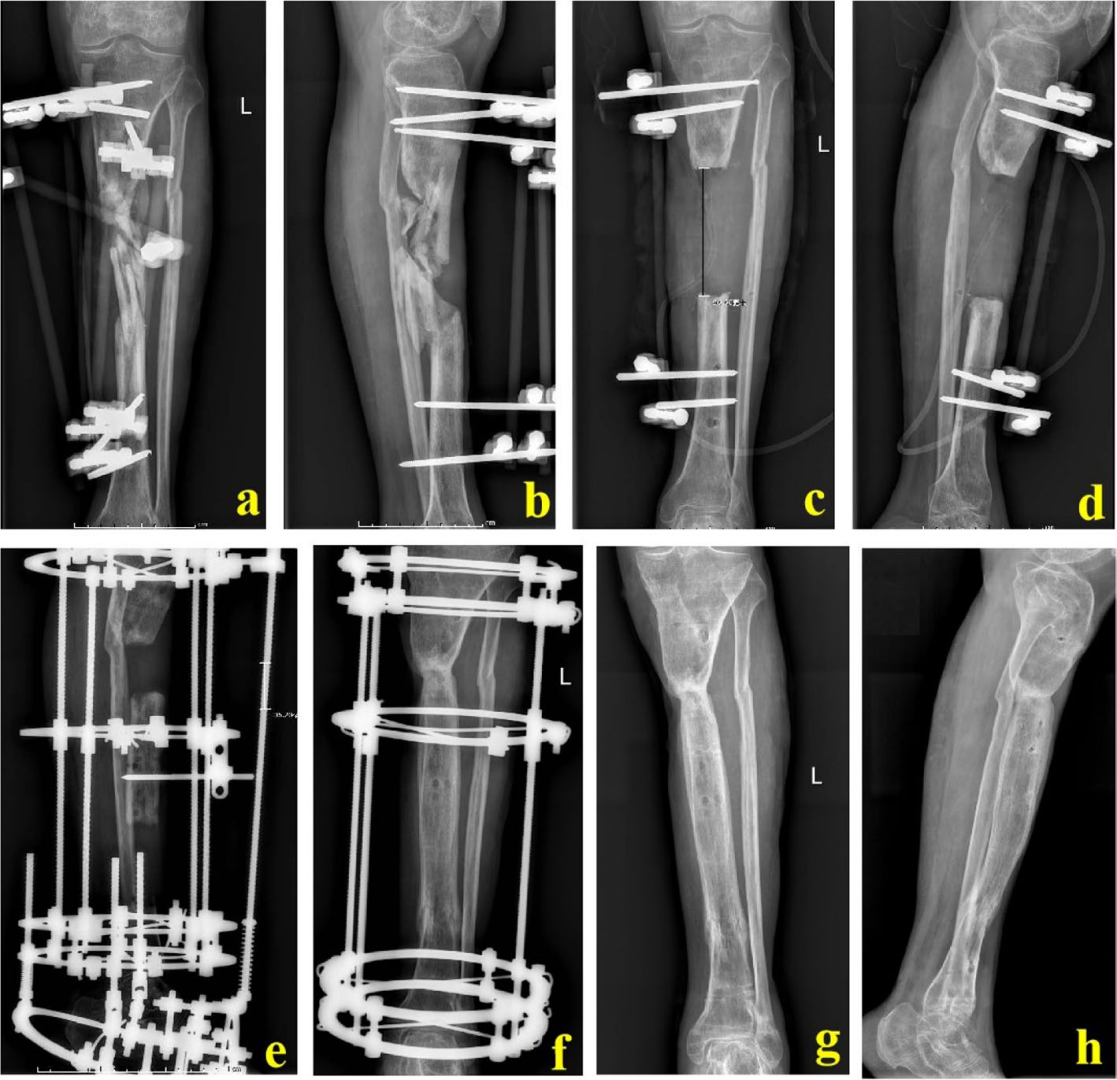
A 53-year-old male was successfully treated by simple bone transport technique. a and b: The patient suffered from a severe open injury and massive sequestrum formed; c and d: After segmental resection of all sequestrum, the bone defect length was 10.5 cm; e and f: Through simple bone transport technique, the bone defects were successfully repaired; g and h: X-ray images after removing the transport frame

For Group B, bone transport process continued until both ends of the bone defects were in contact. Bone ends were pressed properly to make the docking site heal initially. After the docking site firmly healed and the mineralization of the new callus finished, the transport frame could be removed. A typical case was shown in Fig. 4.

### Postoperative treatment

After operation, according to the surgeon’s guidance, the passive and active functional exercises were properly carried out, and symptomatic treatments such as anti-inflammatory, detumescence and pain relief were given. During bone transport process, patients were required to recheck every 2 weeks. X-ray images were taken to evaluate the bone transport status and docking site healing. During the follow-up period, patients were guided to bear weight properly. Surgeons should correct their walking gait and teach them to avoid excessive or insufficient weight-bearing exercises.

### Observation indexes

The follow-up time, time in external fixator, total cure time and number of operations for Group A and B were recorded. Patients were followed through outpatient review, telephone, wechat platform, etc. Limb functions were evaluated by Ennecking score [16], including pain, activity function, self feeling, brace use, walking ability and gait change. Each item was scored 0–5 points and the full score was 30 points. The cumulative score divided by 30 points was the percentage of normal limb function recovery. The anxiety status was evaluated by SAS score [17] which included no anxiety, mild anxiety, moderate anxiety and severe anxiety. Moreover, the total complication incidence was compared between Group A and B.

### Statistical analysis

SPSS23.0 software was used to process data. Kolmogorov Smirnov was used to test whether the measurement data conformed to the normal distribution. The measurement data conforming to the normal distribution were expressed as mean ± standard deviation. Unpaired t-test was used for comparison between Group A and B. Counting data were analyzed by χ^2^ test, *p* < 0.05 was statistically significant.

## Results

The mean age of Group A and B was (37 ± 12) and (36 ± 11) years, respectively. Male number in Group A and B was 26 and 28, and female was 9 and 14. The bone defect length in Group A and B was (13.3 ± 3.3) and (13.7 ± 3.8) cm, respectively. Group A concluded 13 patients with femoral bone defects while 22 for tibia. Group B contained 14 and 28 cases for femur and tibia. In Group A and B, there were 24 and 27 patients caused by acute trauma while 11 and 15 by chronic osteomyelitis. The mean body mass index (BMI) of Group A and B was (23.3 ± 3.6) and (23.7 ± 3.5) kg/m^2^, respectively. In Group A and B, there were 25 and 29 patients who had no medical diseases before operation while 10 and 13 patients with at least one kind of medical disease, such as diabetes, hypertension, coronary heart disease, gastrointestinal diseases, etc. There were 14 cases of closed fracture, 3 cases of GA I, 6 cases of GA II and 12 cases of GA III in Group A while 17 cases of closed fracture, 5 cases of GA I, 7 cases of GA II and 13 cases of GA III in Group B for the initial injury. There was no significant difference in age, gender, bone defect length, location, etiology, BMI, medical diseases and classification between Group A and B (*p* > 0.05, Table 1).

**Table 1:** Demographics of patients

Variable	Group A (*n* = 35)	Group B (*n* = 42)	*p* value
Age (year)	37 ± 12	36 ± 11	0.675
Sex			0.467
Male	26	28	
Female	9	14	
bone defect length (cm)	13.3 ± 3.3	13.7 ± 3.8	0.623
Location of bone defect			0.727
Tibia	22	28	
Femur	13	14	
Etiology			0.692
Acute trauma	24	27	
Osteomyelitis	11	15	
BMI (kg/m^2^)	23.3 ± 3.6	23.7 ± 3.5	0.626
Medical diseases			0.820
None	25	29	
At least one kind	10	13	
classification			
Closed fracture	14	17	0.966
GA I	3	5	0.919
GA II	6	7	0.956
GA III	12	13	0.756

*Notes*: *BMI* stands for body mass index. *GA* stands for Gustilo-Anderson

The average follow-up time in Group A and B was (28.8 ± 2.9) and (29.3 ± 3.0) months, respectively. The time in external fixator of Group A and B was (4.8 ± 1.6) and (18.2 ± 3.9) months (*p* < 0.05, Table 2). The total cure time of Group A and B was (17.6 ± 2.2) and (20.4 ± 2.8) months (*p* < 0.05, Table 2). The mean number of operations in Group A and B was (4.9 ± 1.2) and (4.8 ± 1.0), respectively. Ennecking score in Group A: 26–30 points, 20 cases; 21–25 points, 13 cases; 16–20 points, 2 cases. The mean score was 25.4 points, and the mean recovery of normal functions was 84.7%. Ennecking score in Group B: 26–30, 11 cases; 21–25 points, 22 cases; 16–20 points, 7 cases; 10–15 points, 2 cases. The mean score was 22.7 points, and the mean recovery of normal functions was 75.7%. Ennecking score in Group A was significantly higher than that in Group B (*p* < 0.05, Table 2). In group A, 25 patients had no anxiety and 10 had mild anxiety. In Group B, 7 cases had no anxiety, 21 had mild anxiety and 14 had moderate anxiety. The percentage of anxious patients in Group B was significantly higher than that in Group A (*p* < 0.05, Table 2).

**Table 2:** Clinical effect evaluation of two treatment techniques

Variable	Group A (*n* = 35)	Group B (*n* = 42)	*p* value
Follow-up (month)	28.8 ± 2.9	29.3 ± 3.0	0.461
Time in external fixator (month)	4.8 ± 1.6	18.2 ± 3.9	0.001
Total cure time (month)	17.6 ± 2.2	20.4 ± 2.8	0.001
Number of operations	4.9 ± 1.2	4.8 ± 1.0	0.696
Ennecking score	84.7% (25.4)	75.7% (22.7)	0.001
26–30	20	11	
21–25	13	22	
16–20	2	7	
11–15	0	2	
SAS score			0.001
No anxiety	25	7	
Mild anxiety	10	21	
Moderate anxiety	0	14	
Complications			
Number of patients	4	23	0.001
Number of complications	5	44	0.001

*Notes*: *SAS* stands for self-rating anxiety scale

Four patients in Group A had complications for 5 times, and 23 patients in Group B had complications for 44 times. There were 2 and 9 cases of axial deviation in Group A and B. The axial deviation was adjusted at the same time when the patient in group A was replaced with bone graft and internal fixation. In Group B, the axial deviation was adjusted by another surgery. In Group A, there were 2 patients with poor bone graft healing. After re bone grafting, the bone defects healed smoothly. However, in Group B, there were 15 patients suffering from docking site nonunion. After sclerotic bone resection, scar resection and autologous iliac bone transplantation, the docking site healed well. In Group A and B, there was one and 13 cases of pin-tract infection, respectively. After removing the original pins and replacing with a new one, the pin-tract infection was effectively controlled. Four patients in Group B had mild foot drop after operation, and the symptoms were slightly improved after soft tissue release. Three patients in Group B had partial limitation of knee joint movement. No special treatment was given for this complication.

## Discussion and conclusions

Large segmental bone defects are a hot and difficult problem concerned by trauma surgeons [18]. Bone transport technique has been successfully applied for the management of large segmental bone defects. However, its main shortcoming is the long-lasting consolidation period, which may cause lots of related complications. It is very inconvenient for patients to wear an external fixator for a long time. Therefore, patients’ satisfaction with this technique is not high.

Several scholars have applied different modified techniques in order to reduce the time in external fixator and related complications, such as bone transport over a nail, acute shortening and re-lengthening, bone transport and then nailing [3, 19–25]. However, the technique of bone transport over a nail has a great risk of infection recurrence. Once the infection relapses, it will significantly increase the number of operations and medical expenses. The acute shortening and re-lengthening technique is usually suitable for bone defects of less than 10 cm [3, 22]. In the face of long bone defects, due to the problems of excessive traction of nerves and vessels and difficult shortening of soft tissues, the curative effects of this technique are not accurate [3, 22]. In our study, patients with large bone defects longer than 8 cm were included, and the acute shortening and re-lengthening technique was not applicable for most of our cases.

Bone transport combined with bone graft and internal fixation is a good solution to shorten the time of external fixation and reduce related complications. Emara et al. used bone transport technique to treat patients with tibial infectious nonunion. When there was nonunion at the docking site, the external fixator was removed, replaced with an intramedullary nail and bone graft [23]. Their research showed that this scheme could shorten the time in external fixator and solve the problem of docking site nonunion, but the patient should be informed of the risk of infection recurrence in advance. Lambiris et al. found that it was a good choice to replace the external frame with an intramedullary nail when docking site nonunion, angular deformity of the transport segment or intolerance of the external fixator occurred [24]. A prospective study by Xiayimaierdan et al. displayed that in the treatment of large segmental bone defects, bone graft and internal fixation after bone transport could promote healing, improve joint functions and reduce complications [25]. Our results were similar to the above studies. However, since we removed the transport frame in time when the bone defects were shortened to within the critical-sized bone defects (5 cm), the time of wearing an external fixator would be further shortened compared with the above studies. The reason why we choose 5-cm-length as a critical criterion is that many studies have shown for bone defects within this length, the scheme of non vascularized autologous bone transplantation has definite curative effects and the amount of autologous bones to be taken is appropriate [12–14]. For bone defects beyond this length, autologous bone transplantation is limited, including insufficient materials, bone resorption or necrosis etc. [15].

Our new technique is an improvement for bone transport in essence. It has many advantages. Based on our results, this technique could significantly shorten the time in external fixator. Bone transport includes transport phase and consolidation phase. Usually, the time of consolidation phase is 2–3 times that of transport phase. Patients treated by simple bone transport technique may wear the external frame for 1 year and even 2–3 years. Wearing it may cause great inconvenience to patients’ life. However, using our modified technique, when the bone defects are shortened to less than 5 cm, the transport frame will be removed. Patients only need to wear the external frame for a few months. This greatly shortens the time in external fixator and enables them to return to social activities as soon as possible. In addition, the overall complication incidence was low and the total cure time was relatively short for our modified technique. Most of the postoperative complications are caused by wearing the transport frame for a long time. The unplanned surgeries for these complications will prolong the treatment cycle and increase the number of operations. Moreover, patients using our modified technique achieved better limb functions compared with those using simple bone transport technique. Although the transport frame is fixed away from the injured site, its fixaion pins will pass through muscles and other soft tissues. The traction to the soft tissues will result in adhesion and contracture. The cutting to the skin and soft tissues will cause chronic pain. These factors are not conducive to patients’ postoperative functional exercises. As a result, it may lead to joint stiffness and foot drop, etc. Besides, patients treated by our modified technique had low postoperative anxiety score and were more likely to tolerate the whole treatment course. Although the transport scheme has developed from the uni-focal to bifocal and trifocal, and the time in external fixator is significantly shortened, its basic principles have never changed, including external fixation and distraction osteogenesis. External fixation usually means discomfort. Patients wearing it for a long time are prone to anxiety, depression and poor compliance.

In our study, two patients suffered from poor bone graft healing in the modified technique group. This was because there was partial bone resorption at the bone graft area, resulting in insufficient bone strength after healing. When using this technique, the amount of bone graft should be sufficient. Cancellous bones are better than cortical bones. The metabolic activity of cancellous bones is 8 times that of cortical bones [26]. The bone graft area is moderately elastic and prone to bone revascularization. After replacement with internal fixation and bone graft, we were also worried about infection recurrence. The possible source of infection is mainly latent at the bone defect area or pin-tracts. Therefore, when facing patients with an open fracture or chronic osteomyelitis, it is very important to perform thorough debridement. Compared with local carpet debridement, segmental resection of all infected bones and sequestrum is more thorough. If local infection or suspected infection is found at the bone defect site during bone graft and internal fixation, trauma surgeons should not hesitate to debride and bite off the infected bones again. After the infection is completely controlled, the bone graft and internal fixation surgery could be performed. In addition, when removing the transport frame, the infected necrotic tissues at the pin-tracts should be scraped off. After the pin-tracts heal, sequential bone graft and internal fixation can be performed. These measures can minimize the possibility of infection recurrence.

There are still some deficiencies in this study. The number of patients was small and the follow-up time was short. For patients with tibial defects of poor skin and soft tissue conditions and patients whose skin and soft tissue defects need to be repaired at the same time, our modified technique may not be performed. These patients may be more suitable for simple bone transport technique. This may lead to a certain deviation in this study, but this deviation will not significantly interfere our conclusions. Patients included in this study were those with bone defects greater than 8 cm. When the bone loss is between 5 and 8 cm, the transport phase required is relatively short. Replacing with an internal fixation device will increase costs and the economic burden of these patients. Therefore, it is still worth further research on which treatment scheme can obtain the maximum benefits for these patients.

## Conclusions

Based on our findings, the clinical effects of bone transport combined with bone graft and internal fixation technique were better than that of the simple bone transport technique, including shorter time in external fixator, shorter total cure time, lower anxiety score and better limb functions.

## Data Availability

The data and materials are available from the corresponding author on reasonable request.
